# Predicting Flowering Behavior and Exploring Its Genetic Determinism in an Apple Multi-family Population Based on Statistical Indices and Simplified Phenotyping

**DOI:** 10.3389/fpls.2017.00858

**Published:** 2017-06-07

**Authors:** Jean-Baptiste Durand, Alix Allard, Baptiste Guitton, Eric van de Weg, Marco C. A. M. Bink, Evelyne Costes

**Affiliations:** ^1^Laboratoire Jean Kuntzmann, Inria Mistis, Université Grenoble AlpesGrenoble, France; ^2^Virtual Plants Team, Inria and CIRAD, UMR AGAPMontpellier, France; ^3^Equipe Architecture et Fonctionnement des Espèces Fruitières, UMR AGAP, Institut National de la Recherche AgronomiqueMontpellier, France; ^4^Wageningen UR Plant Breeding, Wageningen University and ResearchWageningen, Netherlands; ^5^Biometris, Wageningen University and ResearchWageningen, Netherlands; ^6^Research & Technology Centre, Hendrix GeneticsBoxmeer, Netherlands

**Keywords:** bayes factor, biennial bearing, entropy, Malus × domestica, markov models, pedigree based analysis

## Abstract

Irregular flowering over years is commonly observed in fruit trees. The early prediction of tree behavior is highly desirable in breeding programmes. This study aims at performing such predictions, combining simplified phenotyping and statistics methods. Sequences of vegetative vs. floral annual shoots (AS) were observed along axes in trees belonging to five apple related full-sib families. Sequences were analyzed using Markovian and linear mixed models including year and site effects. Indices of flowering irregularity, periodicity and synchronicity were estimated, at tree and axis scales. They were used to predict tree behavior and detect QTL with a Bayesian pedigree-based analysis, using an integrated genetic map containing 6,849 SNPs. The combination of a Biennial Bearing Index (BBI) with an autoregressive coefficient (γ_*g*_) efficiently predicted and classified the genotype behaviors, despite few misclassifications. Four QTLs common to BBIs and γ_*g*_ and one for synchronicity were highlighted and revealed the complex genetic architecture of the traits. Irregularity resulted from high AS synchronism, whereas regularity resulted from either asynchronous locally alternating or continual regular AS flowering. A relevant and time-saving method, based on *a posteriori* sampling of axes and statistical indices is proposed, which is efficient to evaluate the tree breeding values for flowering regularity and could be transferred to other species.

## Introduction

Biennial bearing, defined as the irregular fruit or seed production over consecutive years, is a trait commonly observed in perennial crops (Monselise and Goldschmidt, 1982; Samach and Smith, 2013). In fruit trees, yield and fruit quality depend on bearing behavior, which is in turn strongly dependent on flowering intensity. However, floral induction may be inhibited by concurrent fruiting, leading to biennial bearing. Economically and environmentally sustainable techniques are therefore required for the management of biennial bearing in fruit production. An alternative strategy would be to select cultivars combining high fruit quality, long-term resistance to pests and diseases, tree architecture adapted to modern training systems and regular production. Breeding processes in trees are usually slower than in crops, because of the juvenile phase length and the long time required to assess the agronomic performances of pre-selected trees, especially bearing behavior (Laurens et al., 2012). Predicting bearing habit as soon as possible from the beginning of the genotype's production is thus of high interest. This strategy is reinforced by the existence of large differences among cultivars (Lauri et al., 1997, 2014) and the demonstration of genetic control of biennial bearing in an apple family derived from a cross between biennial and regular bearing parents (“Starkrimson® Red Delicious” × “Granny Smith”; SG) (Guitton et al., 2012).

To characterize successive yields and bearing behavior, different approaches have been proposed (see Durand et al., 2013 for a review). The Biennial Bearing Index (BBI; see the list of abbreviations and notations in Table 1), which estimates the intensity of deviation in yields during successive years (Wilcox, 1944), has become the accepted standard to describe biennial bearing. It has been applied to yield (mass of fruit) at different scales: whole areas, individual trees or branches—on apple and other fruit tree species (Pearce and Doberšek-Urbanc, 1967; Reddy et al., 2003; Smith et al., 2004; Rosenstock et al., 2010; Guitton et al., 2012). However, the measure of the magnitude of irregular bearing by BBI is questionable, especially for trended series (Huff, 2001). A new methodology was thus introduced to characterize the bearing habit of trees as early as the first years of production, when the production is increasing. This methodology was based on a trend model on the yearly number of flowers, combined with a BBI-derived index and an index on correlations between residuals, denoted by γ (see Supplementary Material M1). An approximation of these indices based on within-tree sampling of successions of annual shoots (AS) along axes was considered. An entropy criterion was proposed to assess synchronicity of flowering in a given year, allowing a connection between axis- and tree-scale behaviors. However, the ability of axis-scale indices to predict genotype habits at tree scale was investigated on a single family (SG), and the annual shoot sequences were merely used to approximate the total number of flowering AS.

**Table 1:** **Abbreviations used in this article**.

AIC	An (/Akaike) information criterion. Criterion used to select between statistical models (not necessarily nested)
ANOVA	Analysis of variance
AS	Annual shoot
$\text{auto}.\text{cov}\left(\gamma_{g}\right)$	Index quantifying the tendency of residuals from a linear trend models to change signs over years
BBI	Biennial bearing index
BBI_norm	BBI normalized by the sum of yields
BBI_res_norm	BBI on the residuals of a linear trend model, normalized by the sum of yields
BIC	Bayesian information criterion
BLUP	Best linear unbiased predictor (conditional mean of random effects)
Entropy	Indicator of the randomness of some variable (0 corresponding to non-random)
${\bar{\begin{array}{c} Ent \end{array}}}_{g}$	Entropy of flowering probabilities of a given genotype *g*
${\bar{\begin{array}{c} Ent \end{array}}}_{glmm,g}$	Entropy of flowering probabilities issued from some GLMM
η_*g, t*_	Random genotype x year interaction in the Markovian model for the probabilities of flowering
*F*_*g, r*, π, *t*, ℓ_	Presence (1) or absence (0) of flowering for a given genotype, replication, site, year and position on a axis
GLMM	Generalized linear mixed model
HIVW, N, P	Families of apple trees planted at the INRA Angers experimental station, from X3263 × X3259, X3305 × X3259 and “Rubinette” × X3305 cross, respectively (Figure 1)
IBD	Identity by descent
M, A	Montpellier vs. angers (locations of the trees in France)
MCMC	Markov chain monte carlo: simulation algorithm for approximate Bayesian inference
Memory	History of the flowering events related to the current position of an AS within its axis (order 2 memory is the history of the last two events)
NN	Neural network (non-linear regression model)
PBA	Pedigree based analysis
PCA/PC	Principal Components analysis, principal component
QTL	Quantitative trait loci
SG	“Starkrimson^®^ red delicious” × “Granny Smith” family planted in Montpellier (Figure 1)
SNP	Single-nucleotide polymorphism
θ_*g, m*_	Random genotype x memory interaction in the Markovian model for the probabilities of flowering
trait_ax	Trait estimated using axis-scale data (e.g., BBI_res_norm_ax)
trait_pred	Trait at tree scale predicted from axis-scale data and a (non-linear) regression model (e.g., BBI_res_norm_pred)
XB	X3263 × “Belrène” family planted in Montpellier (Figure 1)

*In red, abbreviations used for indices. In blue, abbreviations used for naming apple tree families*.

In the present study, we propose to extend the previous investigations by exploring new methods and indices based on the analysis of sequences of flowering shoots, and by performing a multi-family QTL detection to enlarge the genetic basis of biennial bearing variation in apple trees. We assumed that the analysis of entire sequences of successive AS, combined to flowering synchronicity in each year, would provide new insights on the genotype's behaviors. We thus proposed (i) to use not only the total number of flowering AS but also the vegetative ones and their succession; (ii) to derive new indices from this analysis at no additional measurement cost; (iii) to re-examine previous assumptions on the relation between alternation and regularity at tree and axis scales. Regarding genetics, we considered a larger germplasm to allow the comparison of alleles' performance in different genetic backgrounds (Pauly et al., 2012). We assumed that this will increase the number of segregating QTLs, detection power, accuracy of positions, and give more robust estimation of QTL effects (Bink et al., 2002; Liu et al., 2012). A Pedigree Based Analysis (PBA) was performed, using the concept of Identity By Descent (IBD) based on both pedigree and marker information (van de Weg et al., 2005; Luan et al., 2012). Our aim was to confirm previously found QTLs on the SG family and find new ones, tracing the original source of the favorable alleles and deepening our understanding of the genetic determinisms of biennial bearing in apple tree.

## Materials and methods

### Plant material

**Figure 1:**
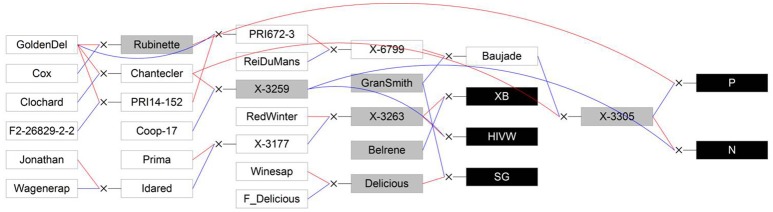
**Genetic relationships between the five studied full-sib families (XB, HIVW, SG, N, and P; represented by black boxes) and their parents (represented by gray boxes) and founders or other members of the pedigree (represented by white boxes)**. Blue lines link the father to its progenies and red lines link the mother to its progenies. GoldenDel, “Golden Delicious,” ReiDuMans, “Reinette du Mans,” Wagenerap, “Wagenerapfel”; see text for family abbreviations and supporting information 2 for other abbreviations used in the pedigree. Reproduced from Allard et al. (2016) with the permission of Oxford University Press.

Five segregating families with known and related pedigree were used (Figure 1). The first family (Segura et al., 2006) is derived from a cross between a female parent (“Starkrimson® Red Delicious”) having strong tendency to biennial bearing and a male parent (“Granny Smith”) prone to regular bearing. This family, hereafter referred as SG, is composed of 123 genotypes, each replicated twice in the same site, and among which 115 individuals were genotyped and phenotyped (Table 2). The second family, referred as XB (Celton et al., 2011), is from a cross between the hybrid X3263 (regular bearer) and “Belrène” (biennial bearer). It comprises 324 genotypes among which 50 were randomly selected for replication, resulting in a total of 374 trees that were all phenotyped, among which 58 were also genotyped. For both families, seedlings were grafted on a semi-dwarfing Pajam I rootstock and planted in 2004 and 2005, respectively, in a random experimental design at the Diascope INRA Montpellier experimental unit. All trees were grown under irrigated conditions with minimal training. In the SG family, branches along the trunk were removed below 50 cm in the first year and fruits were slightly thinned in the first 2 years of growth to avoid branch breaking. In XB family the trees were neither pruned nor the fruits thinned (Table 2).

**Table 2:** **Information on the different families used and sampling strategy**.

**Family**	**Parents**		**Plantation year**	**Site**	**Gen. Nb^a^**	**Rep Nb**	**Management**	**Observation Scale**	**Axis Nb**	**Nb of years**
	**Female**	**Male**								
SG	“Starkrimson® Red Delicious”	“Granny Smith”	2004	Montpellier	115	2	Slightly pruned	Tree	All	6
							Slightly thinned	Axes Trunk	1	6
							Treated and irrigated	Long Sylleptic shoots	1	6
								Long Proleptic shoots	1	6
								Short axillary shoots	18	1–5
XB	X-3263	“Belrène”	2005	Montpellier	58	1	Not pruned	Axes	3^b^	
							Not thinned	Long Proleptic shoots	9^b^	
							Treated and irrigated	Short axillary shoots		
HIVW	X-3263	X-3259	1992	Angers	172	1	Pruned	Axes	9^b^	6–7
										6–7
N	X-3305	X-3259	1992	Angers	42	1	Thinned	Long Proleptic shoots	3^b^	6–7
							Treated and irrigated	Short axillary shoots		6–7
P	“Rubinette”	X-3305	1992	Angers	45	1				

*The columns indicates the names of the family, the names of its parents, the year it was planted, the site where it was planted, the number of genotypes composing the family, the number of replications for each genotype, comments about the orchard management, the observation scales, the number of axes observed per tree, the number of years of the observed shoots*.

a Genotyped and phenotyped;

b *On average*.

The three other families, called HIVW, N, and P, respectively, were chosen for their related pedigree. The HIVW family had a parent (X3263) common to the XB family and the other one (X3259) to the N family. Both N and P families had a common parent (X3305) and the three families derived from “Golden Delicious” with different parentage degrees (Figure 1). They are composed of 171, 42, and 45 individuals, respectively, each with a single replicate per genotype. They were planted at the INRA Angers experimental station, in 1992. The trees were trained in vertical axis with an annual manual thinning that was performed at the end of June and left one fruit per inflorescence. At both sites, pest and disease management was performed consistently with professional practices.

### Phenotyping

On the SG family, as described in Durand et al. (2013), successions of vegetative vs. floral AS were observed over consecutive years (based on the presence/absence of an inflorescence) along different types of axes: trunk, long and short axillary shoots (Table 2). Shoots were classified depending on their length. A distinction was also made between the long proleptic and sylleptic axillary shoots (see Segura et al., 2006). Flowering occurrence was observed along the trunk of each tree, as well as one long sylleptic and one long proleptic axillary shoot, both sampled on the first AS of the trunk (2004 AS). On each long axillary shoot, two short axillary shoots per AS were phenotyped the same way. Thus, 10 short axillary shoots of 5 to 1 years were recorded on long sylleptic, and 8 short axillary shoots of 4 to 1 years were recorded on long proleptic ones. The flowering pattern was described by recording the presence/absence of flowering event on AS (6 possible flowering occurrences on the trunk and long sylleptic shoots, 5 on the long proleptic axillary shoots). The data thus consisted in vegetative vs. floral AS in 6 to 1 year sequences, with 2,716 sequences in total, with a mean length of 3.0 (corresponding to 3 consecutive AS, or years, in average).

For the XB, HIVW, N, and P families, similar observations were performed on three long proleptic axillary shoots along which, four and three short AS were phenotyped along 2006 and 2007 AS, respectively. The numbers of sequences were 7,757 in XB (with mean length of 5.3), 1,511 in HIVW (mean length of 6.0), 442 in P (mean length of 6.2), and 905 in N (mean length of 6.4; see distributions in Supplementary Figure S1).

### Statistical modeling of AS fate sequences

Our approach is based on the classical BBI and on indices defined in Durand et al. (2013): BBI-derived indices (denoted BBI_norm and BBI_res_norm), autoregressive coefficient γ_*g*_ and entropy. They are based on counts of flowering AS at axis and whole-tree scales, whenever possible. The description of the trend and autoregressive models, BBI-derived indices and the statistical methodology for classification of genotype habit can be found in Supplementary Material M1. Compared to the original indices, we added a fixed “site” effect, Montpellier (M) or Angers (A), in the trend and autoregressive models, whenever possible.

At the axis scale, sequences of AS fates are denoted (*F*_*g, r*, π, *t*, ℓ_)_ℓ≥0_ with (*F*_*g, r*, π, *t*, ℓ_ = 0) denoting the absence and (*F*_*g, r*, π, *t*, ℓ_ = 1) the presence of flower for replication *r* of genotype *g* at site π, year *t*, and location (or AS) ℓ in the axis. The indices at axis scale, denoted by BBI_ax, BBI_norm_ax, BBI_res_norm_ax, and γ^*ax*^, were computed as those defined at tree scale but using the total yearly counts of flowering AS in axes sampled within each tree replicate.

These counts were also used to compute two entropies, denoted respectively ${\bar{\begin{array}{c} Ent \end{array}}}_{g}$(entropy based on frequencies) and ${\bar{\begin{array}{c} Ent \end{array}}}_{glmm,g}$ (entropy based on a generalized linear mixed model-GLMM). These two indices are based on the assumption of independent Bernoulli distributions for the successive AS fates *F*_*g, r*, π, *t*, ℓ_. This assumption led us to ignore dependencies and patterns of alternation that could be inferred from sequences of AS fates along axes.

It can be assumed that models and indices taking explicitly into account the succession of AS fates would yield better predictions of genotype habit and provide new insights on the relationship between alternation at axis and whole tree scales. This is why we modeled the sequence of AS fates (*F*_*g, r*, π, *t*, ℓ_)_ℓ≥0_, in this new study, using high-order Markov chains (see Costes and Guédon, 2012, for discussion of the shortcoming of using first-order rather than high-order Markov chains). In such models, the variable at time *t* depends on the *M* past variables where *M* is the order or memory length. The values of these *M* past variables are referred to as the memory *m* of the model at time *t*. For example, memory “10” means that flowering occurred at time *t*–2 but not at time *t*–1. Markov chains with different orders were estimated on the basis of every non-overlapping sequence extracted from the trees. A second-order Markov chain was chosen by a model selection procedure based on the Bayesian Information Criterion (BIC, see Kass and Raftery, 1995). This choice of order means that knowledge of the presence/absence of flowers at years *t*–1 and *t*–2 is necessary for prediction of flowering at year *t*. Thus, the set of memories was {00, 10, 01, 11}.

Assuming that alternation is partly genetic, some interactions between year *t*, memory *m* and genotype *g* should have an effect on flowering. To model these interactions with binary observations *F*_*g, r*, π, *t, m*, ℓ_, approaches based on GLMMs are relevant (Molenberghs and Verbeke, 2005). The following Markovian GLMM was considered:

(1) $$\begin{array}{l} \log\frac{P(F_{g,r,\pi ,t,m,\ell }=1)}{P(F_{g,r,\pi ,t,m,\ell }=0)}=\lambda +\rho_{\pi }+\mu_{m}+\varphi_{t}+\theta_{g,m}\\ \;+\;\eta_{g,t}+\zeta_{g,r}, \end{array}$$

where λ is a fixed intercept, ρ_π_ is the fixed effect of site π (with Angers site as reference ρ_*A*_ = 0), μ_*m*_ is the fixed effect of memory *m* (with reference μ_00_ = 0), φ_*t*_ is the fixed effect of year *t* (with reference φ_2006_ = 0) treated as a qualitative variable, variables θ_*g, m*_ are independent random interactions between genotype *g* and memory *m* with common variance $\tau_{\theta }^{2}$, variables η_*g, t*_ are independent random interactions between genotype *g* and year *t* with common variance $\tau_{\eta }^{2}$, and variables ζ_*g, r*_ are independent replication-specific random effects with common variance $\tau_{\zeta }^{2}$. All random effects were assumed to be mutually independent and Gaussian. This model consists of a high-order Markov chain for process (*F*_*g, r*, π, *t, m*, ℓ_)_ℓ≥0_ where the transitions are treated as GLMMs, so as to introduce fixed and random effects in modeling binary outcomes. Parameter estimation was by restricted maximum likelihood. For a better interpretation of the model and obtain new indices, it is useful to estimate the value of the random effects. This is achieved by their Best Linear Unbiased Predictors (BLUPs), which are the conditional means and also the most probable values of the random effects. These were computed by *glmer* and *ranef* functions of package *lme4* (Bates et al., 2011). The BLUPs of θ_*g, m*_ were used to discriminate genotypes on their low vs. high probability of AS bearing flowers at year *t* given they had memory *m*. Similarly the BLUPs of η_*g, t*_ were used to discriminate genotypes on their low vs. high probabilities of bearing flowers at year *t*.

### Predicting tree fruiting behavior from axis-scale indices

**Figure 2:**
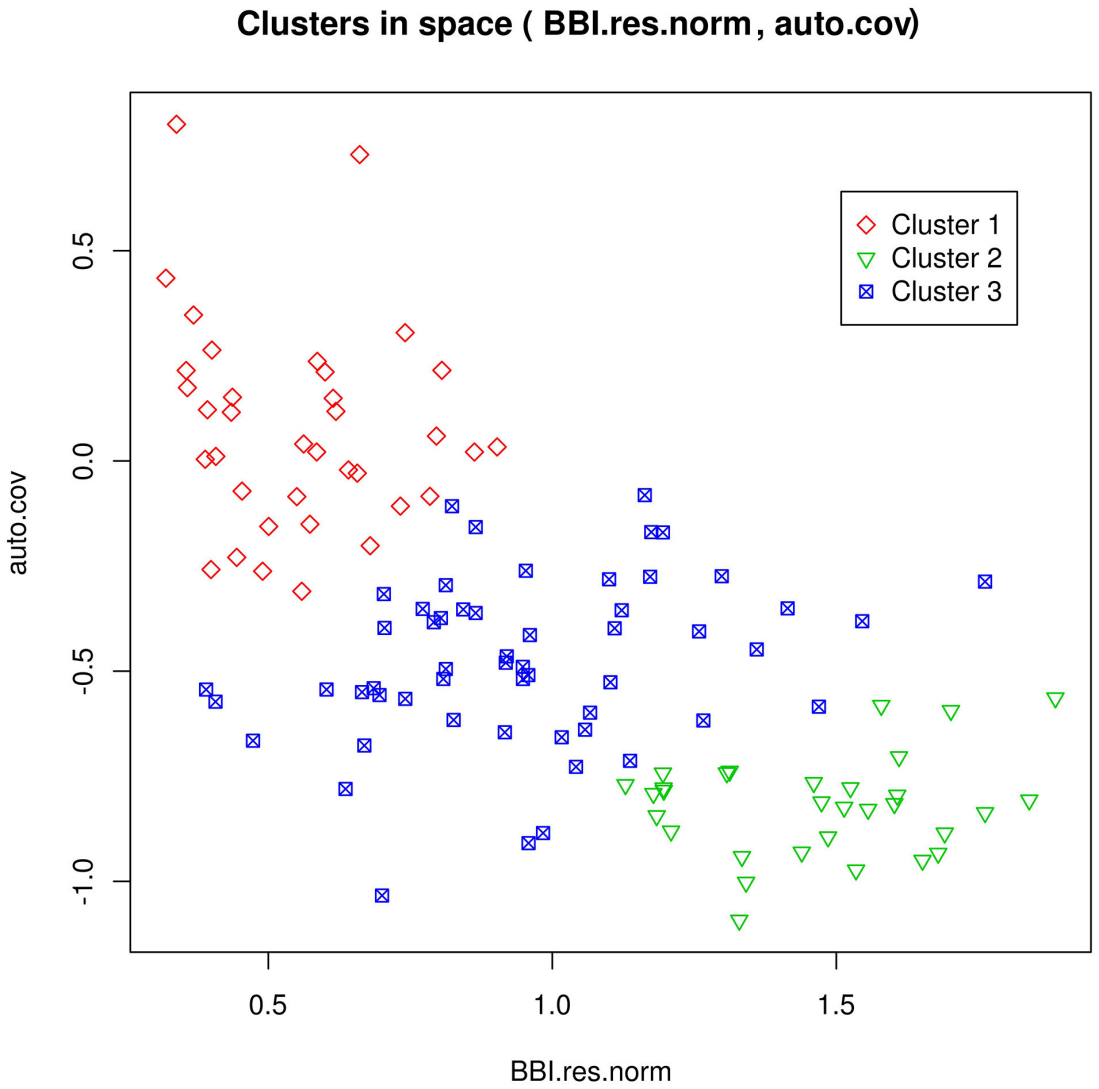
**Representation of the clusters for the genotypes in the SG family, as a function of the tree-scale indices BBI_res_norm and γ_***g***_ (auto.cov)**. Cluster 1 can be interpreted as regular bearing genotypes, cluster 2 as biennial bearing genotypes, and cluster 3 as irregular bearing genotypes. Reproduced from Durand et al. (2013) with permission from Oxford University Press, Copyright 2013.

One goal was the prediction of tree flowering behavior with respect to three classes: regular, irregular or biennial. We assumed that the true class of each genotype could be deduced from the tree-scale indices, for the SG family, using a clustering method developed in Durand et al. (2013). The dependencies between class and tree-scale indices are represented in Figure 2. The classification of the genotypes of all families from axis-scale indices was achieved using as predictors: the BLUPs of the θ_*g, m*_ random effects, BBI_res_norm_ax, γ^*ax*^, ${\bar{\begin{array}{c} Ent \end{array}}}_{g}$and ${\bar{\begin{array}{c} Ent \end{array}}}_{glmm,g}$ (which were available for every genotype, as opposed to whole tree-scale predictors). Note however that BLUPs may not be defined for every parameter and genotype. For example, if memory 11 did not occur in the axes of some genotype *g*, θ_*g*, 11_ cannot be defined, resulting into missing predictors. Moreover, some predictors could be highly correlated and redundant. To handle both issues of predictor absence and redundancy, a principal component analysis (PCA) for partially missing data was used, with the R package missMDA (Josse and Husson, 2012). Classification was performed using neural networks (NNs, see Supplementary Material M2). The number of principal components (PCs) and the NN regularization parameter were determined by out-of-sample validation.

Although classification can be a relevant method to assess regularity at tree scale, its prediction is a rough summary (through three classes only) of the flowering behavior. In other contexts, such as genotype ranking, quantitative assessment of the bearing behavior may be required. This could be achieved through the tree-scale indices BBI_res_norm and γ_*g*_ (when measured), since they provide a more accurate description of this behavior. Since tree-scale indices were known for SG only, approximation of both indices was performed by regression, from axis-scale indices θ_*g, m*_, BBI_res_norm_ax, γ^*ax*^, ${\bar{\begin{array}{c} Ent \end{array}}}_{g}$and ${\bar{\begin{array}{c} Ent \end{array}}}_{glmm,g}$. NNs were used as nonlinear regression functions (instead of nonlinear classifiers in the case of classification), also using missing data PCA. The NN parameters were estimated by least squares minimization. Since the optimal numbers of PCs to be used in classification and regression NNs may be different, they were both chosen independently by out-of-sample validation. The approximated values of BBI_res_norm and γ_*g*_ are referred to as BBI_res_norm_pred and γ^*pred*^, respectively. These two values are necessarily linearly dependent due to the nature of NNs.

Classification and regression were also performed in Durand et al. (2013) but the models did not consider ${\bar{\begin{array}{c} Ent \end{array}}}_{glmm,g}$ nor θ_*g, m*_. Therefore, we assessed the gain of using these new indices as predictors. Since the tree-scale indices were known for SG only, the classification and regression errors were assessed on this family.

### Genetic map and QTL mapping

The five full-sib families and their progenitors were genotyped with the Infinium® 20K SNP array (Bianco et al., 2014), according to Chagné et al. (2012) and Antanaviciute et al. (2012). A genetic map composed of 7,100 SNPs has been integrated over 27 full-sib families using the same approach as in Di Pierro et al. (2016), and was used for QTL mapping (van de Weg et al., unpublished). 6,849 SNPs were used after careful checking of their robustness (van de Weg et al., 2013; Di Guardo et al., 2015), consistency and recombination pattern on the 5 families and the pedigree members (Allard et al., 2016). The quality of the map was achieved by intense data curation and by using graphical genotyping to avoid double recombinations along with the use of multiple families to create an integrated genetic map that reduced cases of false marker order. The high quality of our current map is underlined by the low number of SNPs that are in discordant order (71 SNPs, 3.2%), the small size of the genetic segments in which these discordant orders occurred (usually 0.5 cM, data not shown), and the similar small size of both genetic maps. Then sets of single SNPs were integrated into haploblocks, corresponding to successive 1 cM segments. Haplotypes were composed using the software FlexQTL™ (www.flexqtl.nl) and PediHaplotyper (Voorrips et al., 2016).

Indices used in QTL analysis were BBI_res_norm_ax, γ^*ax*^, BBI_res_norm_pred, γ^*pred*^, ${\bar{\begin{array}{c} Ent \end{array}}}_{g}$, ${\bar{\begin{array}{c} Ent \end{array}}}_{glmm,g}$, the BLUPs for genotypes × memory interactions (θ_*g*, 00_, θ_*g*, 01_, θ_*g*, 10_, θ_*g*, 11_) and genotype × year interactions (η_*g*, 2006_, η_*g*, 2007_, η_*g*, 2008_, η_*g*, 2009_, η_*g*, 2010_, η_*g*, 2011_, η_*g*, 2012_). Among all variables, QTLs were detected using a linear model that comprised an intercept μ, the regression coefficients *a* on the QTL covariates, and a residual *e*, as:

(2) y=μ+Wa+e

where *W* is the design matrix for the QTL effects. A bi-allelic model is assigned to a QTL with alleles denoted by Q and q, with only additive effects and values of [QQ, Qq, qq] equal to [1, 0, −1]. As QTL genotypes of individuals are a priori unknown, modeling is based on independent assignment of alleles to founders and segregation indicators to trace transmission from parents to offspring (Bink, 2002). Uniform priors were assigned to μ and λ (vector giving QTL position), and normal priors to the vectors *a* and *e* in (2), i.e., *a*~$N\left(0,\sigma_{a}^{2}I\right)$ and *e*~$N\left(0,\sigma_{e}^{2}I\right)$. $\sigma_{a}^{2}$ and $\sigma_{e}^{2}$ are the per-QTL explained variance and the residual variance, with priors being inverse Gamma distributions (Bink et al., 2008). The number of QTLs was assigned a Poisson prior. Results for a prior mean of 5 are reported only. Other values yielded similar results and inferences (data not shown). Samples from the joint posterior distribution $f\left(\mu ,a,\lambda ,\sigma_{a}^{2},\sigma_{e}^{2}|y\right)$ of the model parameters were obtained by Markov chain Monte Carlo (MCMC) simulation in FlexQTL™ (Bink et al., 2008, 2014).

The MCMC algorithms and details on the monitoring of Monte Carlo accuracy and length of the simulation chains can be found in Bink et al. (2008) and Allard et al. (2016), respectively. The number of QTLs was inferred from a pairwise comparison of models differing by one QTL, and considering twice the natural logarithm of the Bayes Factors (Kass and Raftery, 1995), denoted 2^*^lnBF. Values >2, 5, and 10 indicate positive, strong, and decisive evidence for the presence of a QTL, respectively. QTL positions were based on posterior QTL intensities, and QTL contributions on the posterior mean estimates of the QTL effects. Posterior probabilities of QTL genotypes were also estimated (Bink et al., 2014).

When several QTLs were detected for a variable, the interactions between QTLs were tested by linear models with haplotypes located at the peak of the QTLs. Model selection was achieved with a backward method based on AIC (Burnham and Anderson, 2002).

## Results

### Modeling as sequence

A Markovian GLMM was estimated merging the five families (1) in Section Statistical Modeling of AS Fate Sequences). It had a BIC value of 44,300. It was compared with the models (i) without any random effect (i.e., with $\tau_{\theta }^{2}=\tau_{\eta }^{2}=\tau_{\zeta }^{2}=0$, BIC = 52,944), (ii) containing “genotype” and “replication” random effects only (no interactions with year or memory, BIC = 50,723), (iii) without “replication” random effects (BIC = 44,394) and (iv) without “genotype” random effects and their interactions (BIC = 50,780). The parameter estimates are in Table 3.

**Table 3:** **Estimates of fixed effects and variances (with the ***p***-values of the tests of the null hypothesis “***n*** = 0” against the alternative “***n*** ≠ 0,” for parameters ***n*** associated with fixed effects) of the mixed model estimating the probability of flowering at axis scale in five apple tree families**.

			**Estimates**	***p*-value**
Intercept		λ	−0.36	0.19
Fixed effects	Site	π_*M*_	−1.05	1e-16
	Memory	μ_10_	1.40	1e-16
		μ_01_	−0.33	1e-07
		μ_11_	0.31	1e-06
	Year	φ_2007_	1.46	1e-07
		φ_2008_	1.81	1e-11
		φ_2009_	1.42	1e-07
		φ_2010_	0.55	0.04
		φ_2011_	1.36	1e-07
		φ_2012_	0.49	0.07
Common variances		$\tau_{\theta }^{2}$	0.54	–
		$\tau_{\eta }^{2}$	2.33	–
		$\tau_{\zeta }^{2}$	0.22	–

*See text for detailed model description*.

The BIC values of these models showed that all fixed (site, memory, year) and the random replication effects were significant, consistently with the associated *p*-values (Table 3). The replication effect, although included in the best model, induced less variability in flowering than memory and year effects (the latter having the largest variance). The trees in Montpellier had lower flowering probability than those in Angers. Flowering AS were more frequent after a vegetative AS preceded by a flowering AS, than directly after a flowering AS (since μ_10_ was higher than μ_01_ and μ_11_), showing frequent biennial alternation in flowering at axis scale. The probability of flowering was the highest in 2008, whereas it was particularly low in 2010 and 2012, whatever the site.

Empirical standard deviations were computed for the BLUPs of random effects θ_*g, m*_ and η_*g, t*_ to estimate their specific variability for each memory *m* or year *t* (Table 4). The random interactions θ_*g*, 10_ (between genotypes and memory “flowering AS followed by a vegetative AS”) had the lowest genetic variability. In contrast, the random interactions θ_*g*, 01_ between genotypes and memory “vegetative AS followed by a flowering AS” had noticeably higher variability. The genetic variability of the random interactions η_*g, t*_ increased with years, showing that the genetic differences became larger with tree age.

**Table 4:** **Empirical standard deviations of random effects for each kind of interaction of the mixed model estimating the probability of flowering at axis scale in five apple tree families**.

**Interactions**	**Std. Dev**.
Genotype × memory	θ_*g*, 00_	0.45
	θ_*g*, 10_	0.35
	θ_*g*, 01_	0.42
	θ_*g*, 11_	0.36
Genotype × year	η_*g*, 2006_	0.81
	η_*g*, 2007_	0.88
	η_*g*, 2008_	1.00
	η_*g*, 2009_	1.09
	η_*g*, 2010_	1.11
	η_*g*, 2011_	1.26
	η_*g*, 2012_	1.73

*See text for detailed model description*.

### Prediction of tree-scale indices using linear regression of axis-scale indices

Trend models of the yearly numbers of flowers at axis scale were used to compute BBI_res_norm_ax and γ^*ax*^ [models (A) and (I) in Supplementary Material M1]. However, the site effect was included in (I) only, since its inclusion in (A) induced non-identifiability issues.

BBI_res_norm and γ_*g*_ were regressed with three or four principal components (PCs) using NNs, and the best cross-validated correlations were obtained with three PCs. The optimal correlation between BBI_res_norm and its prediction BBI_res_norm_pred was 0.71 when using BBI_res_norm_ax, γ^*ax*^ and ${\bar{\begin{array}{c} Ent \end{array}}}_{g}$ as predictors, and 0.72 when using the PCs. The optimal correlation between γ_*g*_ and γ^*pred*^ was 0.60 using BBI_res_norm_ax, γ^*ax*^ and ${\bar{\begin{array}{c} Ent \end{array}}}_{g}$, and 0.64 using PCs. Using ${\bar{\begin{array}{c} Ent \end{array}}}_{glmm,g}$ instead of (or in addition to) ${\bar{\begin{array}{c} Ent \end{array}}}_{g}$ did not improve correlations. Thus, adding information from Markovian GLMMs did not significantly improve the prediction of the tree-scale indices.

### Classification of the genotypes with respect to tree-scale bearing habit

Classification of the genotypes of SG, which may be interpreted as the expected error rate on other families, yielded a 39% cross-validated error rate when using BBI_res_norm_ax, γ^*ax*^ and ${\bar{\begin{array}{c} Ent \end{array}}}_{g}$, and 35% with PCs. Genotypes with unknown values of BBI_res_norm_ax, γ^*ax*^ or ${\bar{\begin{array}{c} Ent \end{array}}}_{g}$were excluded, thus keeping 115 genotypes in SG. As previously, the best prediction was achieved with three PCs. Both ${\bar{\begin{array}{c} Ent \end{array}}}_{glmm,g}$ and ${\bar{\begin{array}{c} Ent \end{array}}}_{g}$ were included in the PCA, in addition to the other axis-scale indices. Although limited, the improvement of the error rate, was significant at level 0.7% on 50 random test samples (Student's *t*-test).

To predict the unknown bearing habits at tree-scale of the genotypes of XB, HIVW, N, and P, the optimal NN model on SG was re-estimated on the whole data set, using genotypes with known classes (i.e., 122 genotypes in SG) for learning the mapping between local indices and classes. Confusion between classes concerned irregular genotypes that could hardly be discriminated from regular and biennial bearing genotypes (Table 5). This comes from irregular genotypes having intermediate values of their indices, between those for the regular and biennial bearing genotypes. As a result, 15 regular and 9 biennial genotypes were classified as irregular, and 9 irregular genotypes were classified as regular. In contrast, one misclassification only occurred between regular and biennial bearing genotypes, highlighting that discrimination between both behaviors is easy.

**Table 5:** **Contingency table for the number of genotypes of each possible true class (corresponding to observations on SG family) assigned to each possible predicted class by NN on local indices**.

		**Predicted class**		
		**Regular**	**Biennial**	**Irregular**
True class	Regular	20	1	15
	Biennial	0	22	9
	Irregular	9	6	40

*For example, among the 36 regular genotypes, 15 were predicted as irregular*.

The three classes were discriminated by analyses of variance (ANOVAs) performed on the axis-scale indices (see Supplementary Table T1). The lowest *p*-values (<1e-8) were obtained with θ_*g*, 01_, ${\bar{\begin{array}{c} Ent \end{array}}}_{g}$, BBI_res_norm_ax and γ^*ax*^, which pointed out especially contrasted values of these indices among the three classes. These four indices thus highlight a higher potential than the other ones to discriminate between classes of bearing behavior.

The model yielded the following predictions:

- SG: 29 (24%) regular, 29 (24%) biennial bearing, 64 (52%) irregular genotypes—with 33% error rate on the learning sample (37% if using BBI_res_norm_ax, γ^*ax*^ and ${\bar{\begin{array}{c} Ent \end{array}}}_{g}$ only)
- XB: 48 (17%) regular, 69 (25%) biennial bearing, 158 (57%) irregular genotypes
- HIVW: 38 (22%) regular, 23 (14%) biennial bearing, 109 (64%) irregular genotypes
- N: 31 (31%) regular, 9 (9%) biennial bearing, 60 (60%) irregular genotypes
- P: 10 (19%) regular, 4 (8%) biennial bearing, 39 (74%) irregular genotypes.

### Consistency of the indices between tree and as scales

Correlation matrices were computed between the tree and axis-scale indices for SG (Table 6) and between indices at axis-scale only for the five combined populations (Table 7). The strongest correlations between tree-scale and axis-scale indices were the correlations between BBI_res_norm_ax, γ^*ax*^ and θ_*g*, 01_ (which correlation is negative with BBI_res_norm_ax and positive with γ^*ax*^). Moreover, θ_*g*, 11_ and ${\bar{\begin{array}{c} Ent \end{array}}}_{g}$ were moderately correlated with BBI_res_norm (negatively) and with γ^*ax*^ (positively), while ${\bar{\begin{array}{c} Ent \end{array}}}_{glmm,g}$ was poorly correlated and θ_*g*, 10_ uncorrelated (at level 0.05) with the tree-scale indices.

**Table 6:** **Correlation coefficients between indices at whole tree scale and indices and BLUPs at axis scale, with 95% confidence intervals, in the SG family**.

		**Tree scale indices**	
		**BBI_res_norm**	**γ_*g*_**
Tree scale indices	γ_*g*_	−0.66 (−0.75, −0.54)	1
Axis scale indices and BLUPs	BBI_res_norm_ax	0.72 (0.61, 0.80)	−0.61 (−0.72, −0.49)
	γ^*ax*^	−0.55 (−0.67, −0.41)	0.51 (0.36, 0.63)
	${\bar{\begin{array}{c} Ent \end{array}}}_{g}$	−0.46 (−0.59, −0.30)	0.34 (0.17, 0.49)
	${\bar{\begin{array}{c} Ent \end{array}}}_{glmm,g}$	−0.19 (−0.36, −0.01)	0.09 (−0.09, 0.27)
	θ_*g*, 01_	−0.55 (−0.66, −0.41)	0.50 (0.35, 0.62)
	θ_*g*, 11_	−0.21 (−0.40, −0.01)	0.17 (−0.03, 0.36)
	θ_*g*, 00_	0.23 (0.04, 0.39)	−0.22 (−0.39, −0.04)
	θ_*g*, 10_	0.00 (−0.21, 0.21)	−0.07 (−0.28, 0.14)

**Table 7:** **Correlation coefficients between the genotype × memory interactions (θ_***g,m***_) BLUP indices and the other indices and BLUPs at axis scale, with 95% confidence intervals, combining the five apple tree families**.

	**γ^*ax*^**	**${\bar{Ent}}_{g}$**	**${\bar{Ent}}_{glmm,g}$**	**Genotype × memory 01 θ_*g*, 01_**	**Genotype × memory 11 θ_*g*, 11_**	**Genotype × memory 00 θ_*g*, 00_**	**Genotype × memory 10 θ_*g*, 10_**
BBI_res_norm_ax	−0.66 (−0.70, −0.62)	−0.28 −(0.35, −0.21)	−0.42 (−0.48, −0.36)	−0.45 (−0.51, −0.39)	−0.38 (−0.44, −0.32)	0.07 (−0.01, 0.15)	0.23 (0.16, 0.30)
γ^*ax*^	1	0.12 (0.05, 0.19)	0.23 (0.16, 0.30)	0.44 (0.38, 0.50)	0.37 (0.30, 0.43)	−0.19 (−0.26, −0.11)	−0.31 (−0.38, −0.24)
${\bar{Ent}}_{g}$		1	0.35 (0.28, 0.41)	0.17 (0.10, 0.24)	−0.16 (−0.24, −0.09)	0.10 (0.02, 0.18)	−0.24 (−0.31, −0.16)
${\bar{Ent}}_{glmm,g}$				0.11 (0.03, 0.18)	0.01 (−0.06, 0.09)	−0.09 (−0.17, −0.01)	−0.14 (−0.21, −0.07)
θ_*g*, 01_				1	0.10 (0.03, 0.18)	−0.37 (−0.44, −0.30)	−0.39 (−0.46, −0.33)
*θ_*g*, 11_*					1	−0.39 (−0.46, −0.32)	−0.24 (−0.31, −0.17)
*θ_*g*, 00_*						1	−0.12 (−0.20, −0.04)

### QTL mapping

**Figure 3:**
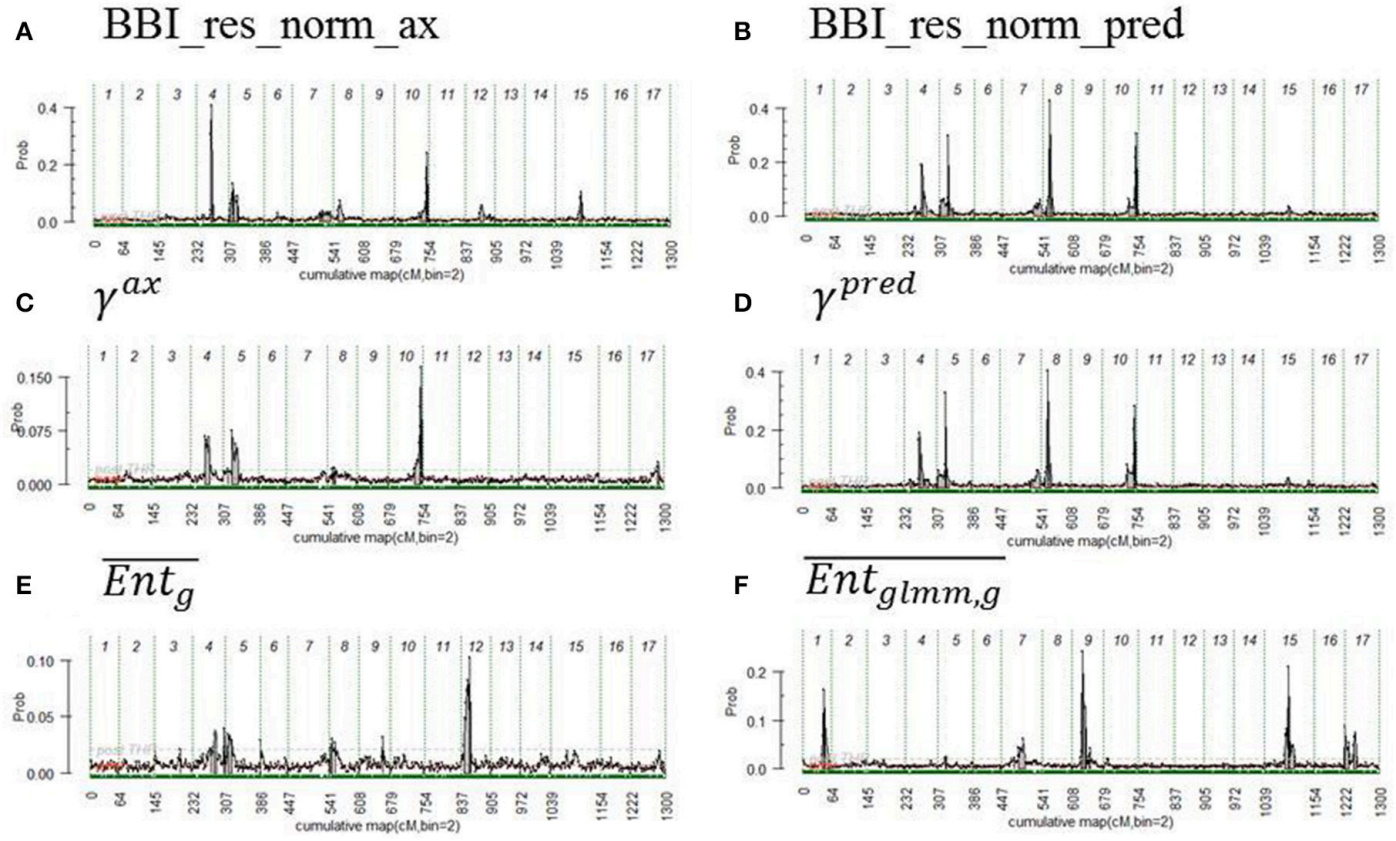
**Posterior probability of QTL position along genome, the beginning and the end of the chromosomes are represented by vertical dashed lines**. The variables displayed are **(A)** BBI_res_norm_ax, **(B)** BBI_res_norm_pred, **(C)** γ^*ax*^, **(D)** γ^*pred*^, **(E)** ${\bar{Ent}}_{g}$, **(F)** ${\bar{Ent}}_{glmm,g}$. See text for abbreviation meaning.

QTLs were detected for all indices except for θ_*g*, 10_, η_*g*, 2007_, η_*g*, 2009_, η_*g*, 2010_, η_*g*, 2011_, and η_*g*, 2012_. Two major QTLs were detected with a strong evidence (2^*^lnBF ≥ 5) on LG4 and LG, for the two BBI-derived indices (BBI_res_norm_ax, BBI_res_norm_pred) (Table 8, Figures 3A,B, Figure S2 in Supplementary Material for the trace plot of QTL across iterations). The QTL detected on LG4 explained 11.5, and 13.3% of variance, respectively. The QTL on LG5 explained 6.9, and 8.3% of the variance of each index, respectively (Table 8). Two other QTLs were detected on LG8 and LG10 but had a strong evidence for BBI_res_norm_pred only. They explained 11.7 and 10% of variance of this index, respectively.

**Table 8:** **Parameters associated with the QTL detected for BBI derived indices, autoregressive coefficients and entropy**.

	**LG**	**2lnBF_LG**	**max_2lnBF_bin**	**pos (cM)**	**Peak (cM)**	**add_ef**	**fq**	**%var**
BBI_res_norm_ax	**4**	**10.6**	**8.94**	**32–41**	**36–37**	**0.39**	**0.12**	**11.5**
	**5**	**5**	**6**	**3–24**	**9–10**	**0.19**	**0.5**	**6.9**
	7	1.9	3.1	65–90	71–72	0.18	0.69	5.4
	8	2.1	4.6	10–23	14–15	0.2	0.39	7.7
	10	4.4	7.4	57–78	75–76	0.21	0.58	7.7
	12	2	4.1	34–43	38–39	0.18	0.39	5.8
BBI_res_norm_pred	**4**	**6.3**	**6.8**	**32–47**	**36–37**	**0.13**	**0.35**	**13.3**
	**5**	**5.8**	**8**	**3–24**	**21–22**	**0.1**	**0.56**	**8.3**
	**8**	**8.4**	**9**	**8–23**	**14–15**	**0.12**	**0.42**	**11.7**
	**10**	**9.3**	**8**	**57–78**	**75–76**	**0.11**	**0.52**	**10**
γ^*ax*^	4	2.7	4.4	34–45	34–35	0.15	0.59	5.3
	5	2.6	4.7	21–36	21–22	0.18	0.41	5.3
	10	3.1	6.4	59–76	75–76	0.18	0.45	5.3
γ^*pred*^	**4**	**6.7**	**6.8**	**32–45**	**36–37**	**0.11**	**0.64**	**12**
	**5**	**6.2**	**8.3**	**3–30**	**21–22**	**0.1**	**0.42**	**10**
	**8**	**9.4**	**8.9**	**8–23**	**14–15**	**0.11**	**0.59**	**12**
	**10**	**9.5**	**7.8**	**57–78**	**75–76**	**0.1**	**0.5**	**10**
*Ent*_*g*_	12	3.1	5.4	6–23	20–21	0.05	0.43	5
*Ent*_*glmm, g*_	1	3.7	6.4	44–59	48–49	0.05	0.63	10
	7	2.4	4.3	39–56	51–52	0.05	0.54	10
	**9**	**8**	**7.4**	**19–34**	**25–26**	**0.07**	**0.75**	**20**
	15	4.5	7.1	46–69	56–57	0.05	0.65	10
	17	3.1	5	0–27	0–1	0.05	0.5	10

The first column indicates the variable concerned, the following columns indicate the LG where the QTL is located, 2ln(BF) value at LG scale, 2ln(BF) value at bin scale, the position of the QTL in cM, the position of the QTL peak, its additive effect, the frequency of positive allele and percentage of variance explained, respectively. Only 2lnBF values corresponding to the comparison of a model with 0 QTL to a model with 1 QTL are presented. QTLs that appear in bold are QTL with a strong evidence for presence, i.e., with a 2^*^lnBF value higher than 5.

Two QTLs were detected on LG8 and LG10 but had a strong evidence for BBI_res_norm_pred only. They explained 11.7 and 10% of variance of this index, respectively.

For the autoregressive coefficients, the same regions along the genome were detected. For γ^*ax*^, three QTLs were detected on LG4, LG 5 and LG 10 (Figure 3C) but none with a strong evidence (Table 8). The QTLs on LG5 and LG10 colocalized with that of the BBI indexes (Figure 3). Four QTLs were mapped for γ^*pred*^ on LG4, LG5, LG8 and LG10 (Figure 3D) and colocalized with BBI_res_norm_pred. QTLs on LG4 and LG8 explained 12%, and those on LG5 and LG10 explained 10% of the variance of γ^*pred*^ (Table 8). Five QTLs were detected for ${\bar{\begin{array}{c} Ent \end{array}}}_{glmm,g}$ (Figure 3F), among which only that on LG9 had a strong evidence and explained 20% of the variance.

The QTL detected for θ_*g*, 00_, θ_*g*, 01_ and_θ_*g*, 11__ (Table T2; Figures S3, S4 in Supplementary Material) on LG10 colocalized with that detected for BBIs. It explained 10, 11.8, and 10.9% of the variance, respectively, and had a strong evidence for θ_*g*, 00_ and θ_*g*, 01_. Two QTLs with positive evidence were mapped on LG12 and LG6, for η_*g*, 2008_ and η_*g*, 2006_, respectively (Table T2; Figures S3, S4 in Supplementary Material).

No interaction between QTLs could be identified for BBI_res_norm_ax and γ^*ax*^, whereas interactions were identified for BBI_res_norm_pred between QTLs on LG4 and LG8, and for ${\bar{\begin{array}{c} Ent \end{array}}}_{glmm,g}$ between QTLs on LG7, LG9, and LG17, with all 2-way and 3-way-interactions being significant (results not shown).

### Genotype estimation at main QTLs

Genotype estimation at QTLs brought two types of information. Firstly, the allelic classes of parents allowed identifying in which family QTLs segregated: if one parent of a family was estimated to be heterozygous at a QTL, this family segregated for this QTL. Secondly, genotype estimation allowed identifying parents and founders bearing favorable alleles, specific to the considered variable. Hereafter, only QTLs with strong evidence were commented (Table 9).

**Table 9:** **Parent genotype estimation for BBI_res_norm_ax at each QTL: qq for homozygous with low value favorable allele, QQ for homozygous with high values unfavorable allele, and Qq for heterozygous**.

	**LG4**	**LG5**	**LG7**	**LG8**	**LG10**	**LG12**	**Nb favorable. alleles**.
X-3305	**qq**	**Qq**	QQ	qq	QQ	qq	7
X-3263	**qq**	**??**	QQ	qq	QQ	??	3
X3259	**qq**	**Qq**	**Qq**	qq	**Qq**	qq	9
“Rubinette”	**qq**	**qq**	??	qq	??	qq	8
“Granny Smith”	**qq**	**QQ**	QQ	qq	QQ	**Qq**	5
“Red Delicious”	**Qq**	**Qq**	QQ	**Qq**	QQ	??	3
“Belrene”	**qq**	**??**	QQ	qq	**Qq**	??	5
“Winesap”	**Qq**	**QQ**	**QQ**	**QQ**	**QQ**	**??**	1
“Wagenerap”	**qq**	**??**	**QQ**	**QQ**	**??**	**??**	2
“ReiDuMans”	**qq**	**??**	**QQ**	**??**	**??**	**??**	2
“RedWinter”	**qq**	**??**	**QQ**	**qq**	**??**	**??**	4
“Prima”	**qq**	**??**	**QQ**	**??**	**??**	**??**	2
“Jonathan”	**qq**	**??**	**??**	**qq**	**??**	**??**	4
“Golden Delicious”	**qq**	**qq**	**QQ**	**qq**	**??**	**qq**	8
F2-26829-262	**qq**	**??**	**??**	**??**	**??**	**??**	2
F_Delicious	**qq**	**qq**	**??**	**qq**	**??**	**??**	6
“Cox Orange”	**qq**	**qq**	**??**	**qq**	**??**	**??**	6
“Coop17”	**qq**	**qq**	**qq**	**qq**	**qq**	**qq**	12
“Clochard”	**qq**	**QQ**	**QQ**	**qq**	**QQ**	**qq**	6

*The favorable allele corresponds to the allele linked to small phenotypic value. The last column contains the count of favorable alleles over all the QTLs. QTLs with strong evidence are identified by a bold font*.

The estimated genotypes were identical for all QTLs that colocalized for the normalized BBI indexes, except for that on LG5 for BB_res_norm_pred (Figure 3). The LG4 QTL segregated in SG only and was heterozygous for “Red Delicious.” The other parents were estimated to be homozygous for the low value allele. As low BBI values indicate a regular bearing habit, “Red Delicious” is likely to transmit the favorable allele to half of its progenies, whereas the other parents are supposed to transmit favorable alleles only.

The QTL on LG5 segregated in all families except XB with parents X-3305, X-3259, and “Red Delicious,” which is estimated to be heterozygous. However, for BBI_res_norm_pred, X-3259 was estimated to be homozygous (Table 9), this questioning the presence of favorable allele at that position. “Rubinette” was estimated to be homozygous for the low value allele therefore supposed to transmit only favorable allele. “Granny Smith” was estimated to be homozygous for the high value allele therefore supposed to transmit unfavorable alleles only.

Three parents, “Rubinette,” “Granny Smith,” and “Belrene,” were estimated to be heterozygous at the LG10 QTL, consistently with the contribution of several families to this QTL (Figure 3). Unstable results were found for the genotype estimation of X-3263, depending on the index used.

“Granny Smith” was estimated to be heterozygous on the LG9 QTL for ${\bar{\begin{array}{c} Ent \end{array}}}_{glmm,g}$, consistently with the segregation of this QTL in SG only. The other parents were estimated to be homozygous for the high value allele (Figure 3). Since individuals with the most regular fruiting behavior had a high value of entropy, these homozygous parents are supposed to transmit favorable alleles.

The genotype estimates over all QTLs for a given trait provide information on the value of the parents and founders as genitors. Taking BBI_res_norm_ax as an example, antagonisms between QTLs were revealed: the parents estimated to be homozygous for the favorable low value allele on LG4 were estimated to be almost systematically homozygous for the unfavorable allele on LG7 (Tables 8, 9). The count of favorable alleles over the six QTLs revealed that the founder “Coop17” and the parent X-3259 had the highest values (12 and 9 favorable alleles, respectively). In spite of QTLs for which no genotype could be estimated, “Rubinette” and “Golden Delicious” appeared interesting parents with homozygous estimated genotypes with the favorable allele at four QTLs over six (Table 9).

## Discussion

### Efficiency of indices derived from whole as sequence analyses at tree scale

In this study, we estimated several indices to capture the bearing behavior of a large set of genotypes. As previously underlined (Durand et al., 2013), BBI_res_norm and γ_*g*_ are negatively correlated and complement one another: BBI_res_norm distinguishes between regular individuals, with low values, and irregular and biennial bearing individuals, with high values. As a complement, γ_*g*_ distinguishes biennial bearing individuals, with negatives values from regular and irregular individuals. The new indices θ_*g, m*_ and their correlations with γ_*g*_ and BBI_res_norm provide complementary information: the regular genotypes (lowest values of BBI_res_norm) exhibit AS with a flowering probability above average at year *t* after flowering at year *t*–1 (highest θ_*g*, 01_ and θ_*g*, 11_, associated with memories 01 and 11). In contrast, θ_*g*, 00_ and θ_*g*, 10_ are less discriminant, because the probability to flower after vegetative events is always high. The positive correlation of entropy ${\bar{\begin{array}{c} Ent \end{array}}}_{g}$ with γ_*g*_ and its negative correlation with BBI_res_norm show that the genotypes with the highest synchronism (lowest value of entropy) are mostly biennial bearers (high values of BBI_res_norm, low values of γ_*g*_).

The correlations between tree- and axis-scale descriptors suggest that biennial bearing at tree scale results from the conjunction of two phenomena: synchronism in flowering between AS in a given year and biennial alternation at AS scale between consecutive years. On the contrary, regularity at tree scale results from either asynchronous locally alternating flowering or regular flowering at AS scale. Irregular genotypes exhibit intermediate values for every descriptor, suggesting that these genotypes are characterized by partial biennial alternation at AS scale or strong biennial alternation with partial synchronism. However, more complex within-tree organization of synchronisms could exist (Couranjou, 1983), that have not been investigated herein. The regular genotypes exhibiting synchronized and regular flowering AS or desynchronized flowering AS will require further investigations regarding fruit set and quality. Indeed, high flowering rate is usually associated with poor fruit set due to environmental (Tustin et al., 2012) or genetic co-variation (Celton et al., 2014). Tree management includes a number of practices to reduce crop load, such as thinning (manual or chemical) or the manual removal of fruiting spurs (Lauri et al., 2007; Breen et al., 2016). We can thus suspect that selecting genotypes with regular desynchronized axes could be an appropriate strategy for avoiding poor fruit set while reducing thinning or manipulations costs. In conclusion, three indices can be considered as key and complementary descriptors of the bearing behavior of genotypes at either tree or axis scales: BBI_res_norm, γ_*g*_ and entropy. The first two are sufficient to classify the genotypes into regular, irregular and biennial classes. However, entropy allows this diagnostic to be refined by providing information on the within-tree strategy of regular genotypes, with potential consequences on tree management and breeding goals.

More insight on the bearing behavior is also gained by introducing site and year effects and analyzing the genotype × year interactions (η_*g, t*_ indices). The lower flowering probability at Montpellier than Angers may result from the absence of thinning practices on XB family, which may have hampered tree flowering capacity over years (Dennis and Neilsen, 1999). However, thinning was performed quite lately in Angers, due to a large dispersion of phenological stages among genotypes (Allard et al., 2016). This practice likely had a relatively low impact on floral induction which is assumed to occur mid-June in apical meristems of spurs (Hanke et al., 2007) and therefore on alternation. Even though the mean probability of flowering of all genotypes per site highlighted phase opposition in the last three years (2010–2012, Supplementary Figure S5, left), no clear characterization of years as being “ON” or “OFF” could be made on the mean values per family (Supplementary Figure S5, right). Thus, no climatic year could be considered as “ON” or “OFF.” Even though critical climatic conditions such as frost (Nagy et al., 2010) or crop load management (Girona et al., 2010) can synchronize trees in a given year and site, different genotypes can be in phase opposition for flowering in a given year, in apple (Durand et al., 2013) as in olive tree (Ben Sadok et al., 2013).

The new indices at axis scale were expected to improve prediction of bearing habits at tree scale. Even though the predicted BBI_res_norm and γ_*g*_ appeared robust based on QTL detection, only a 4% improvement in predictions on test samples was obtained compared with Durand et al. (2013) and the classification error was still of 35% (even though that on SG family was reduced from 37 to 33%). The misclassification mainly concerned the irregular genotypes, whereas the regular and biennial behaviors could be predicted with good accuracy. The misclassification of regular genotypes considered as irregular (15 over 36, see Table 5) could lead to discard them during the selection process. However, this type of error is less problematic than the reverse (selecting irregular genotype that would be misclassified as regular) especially if we consider the drastic reduction in the number of individual selected in the early stages of breeding process. Therefore, the simplified phenotyping strategy that consists in sampling axes within the tree structure with an *a posteriori* observation appears to be relevant. Indeed, this is a time-saving strategy for phenotyping that can be combined to computation of indices for rejecting biennial and irregular genotypes during the assessment of agronomic performance of pre-selected genotypes in breeding programmes. It could also enable further phenotyping of germplasm toward the implementation of DNA-informed breeding approaches by further enlarging the number of founders and breeding parents for which QTL-genotypes are known, or by implementing genomic selection. Both applications are likely to accelerate the breeding progress and overcome long generation intervals and extensive phenotyping in outbred fruit tree crops (Kumar et al., 2013; Muranty et al., 2015).

Moreover, this strategy could be directly used on any species for which retrospective phenotyping of flowering is possible at AS scale. This is the case for species with terminal flowering such as pear, walnut, avocado, mango, litchi etc. in which flowering events can be easily identified. For such species, the methodology proposed, including prediction of flowering behavior at tree scale from *a posteriori* observations and computation of indices would be transposable. For other species, BBI_res_norm and γ_*g*_ index could be computed based on counting the total number of inflorescences measured on several successive years. Even though more time-demanding than retrospective observations, such counts may be facilitated and automatized by new technologies based on imagery (Aggelopoulou et al., 2010; Gongal et al., 2016).

### Genetic determinisms of bearing behavior in a multi-family population

Five major QTLs were yielded, four for BBIs and auto-correlation coefficient (γ_*g*_), on LG4, 5, 8, and 10, and one for entropy on LG9, which were partially common with previous studies on the SG family, such as the QTL on LG4 previously detected for BBI (Guitton et al., 2012). Also, a QTL on LG8 was found for BBI at both tree and axis scales in Durand et al. (2013) and for BBI, yield and number of flowers per inflorescence in Guitton et al. (2012). This zone, located at 8–23 cM on LG8, partially overlapped with QTLs detected in SG family for a descriptor of tree vegetation density (Virlet et al., 2015), for traits linked to bud break (Celton et al., 2011; Allard et al., 2016), and traits involved in gas exchange and xylem conductance (Segura et al., 2008; Regnard et al., 2009; Lauri et al., 2011). The QTL on LG10 located between 55 and 78 cM for BBIs, γ^*ax*^, θ_*g*,00_ and θ_*g*,01_ co-localized with those detected for BBI, precocity and number of seeds per inflorescence in SG (Guitton et al., 2012) and for the percentage of bourses with one fruit on short axes in XB family (Celton et al., 2014). The QTLs detected on LG1 and LG14 by Durand et al. (2013) were confirmed, but in year specific interaction only, whereas the QTL on LG11 could not be confirmed. Actually, re-analyzing the same dataset led us to found an inappropriate account for missing flowering AS that had led to a false QTL detection. This was corrected in the present study.

As previously suggested (Bink, 2002; Liu et al., 2012), a higher power of detection was obtained in a multi-family context, which brought a higher number of segregating regions, alleles and individuals. The QTL on LG9 for entropy appeared as a new zone of importance, as it co-localized with a major QTL detected for the timing of vegetative and flowering bud break (Dyk et al., 2010; Celton et al., 2011; Allard et al., 2016). New QTLs were also detected for BBIs on LG5 and LG7, consistently with “Starkrimson® Red Delicious” and “Granny Smith” not being heterozygous for these QTLs. The QTL on LG5, located from 9 to 24 cM, co-localized with QTLs previously detected for variables linked to the tree fruiting capacity (number of fruits and fruit biomass) under soil water restriction (Virlet et al., 2015). Moreover, LG5 is homologous of LG10 (Velasco et al., 2010; Bushakra et al., 2012), also involved in the fruiting capacity of the trees. As LG5 and LG10 are full-length homologs which orientation is defined upside-down, these two QTLs may have a common underlying mechanism. Altogether these co-localizations suggest that both tree development (LG8) and fruiting capacity (LG5 and LG10) may contribute to the genetic variation of biennial bearing behavior in apple tree. They reinforce previous assumptions regarding the combined effects of both competition among organs for nutrients and hormonal signals on biennial bearing (e.g., Chan and Cain, 1967; Dennis and Neilsen, 1999). To further decipher the putative role of fruits and carbon economy on the inhibition of floral induction, the tools defined herein could be wisely used to classify genotypes before investigating their physiological behaviors.

The lack of QTL interaction detected for BBI_res_norm_ax and γ^*ax*^ suggests that QTL contribution to the genetic variance was properly estimated for these variables. However, the QTL interactions detected for BB_res_norm_pred and ${\bar{\begin{array}{c} Ent \end{array}}}_{glmm,g}$ suggest that the QTL contribution might be underestimated. Taking into account epistasis might provide a better understanding of the genetic architecture of these traits. The complex genetic architecture of the studied traits and the alleles present on the different QTLs may lead to different degrees of alternation. Further characterization of allelic variations will be necessary for analyzing their relative contribution to the tree phenotype.

Finally, no co-localization was found with QTLs detected for architectural traits measured in the first years of tree development (Segura et al., 2007, 2009). Even though qualitative notations of architectural traits collected on young trees and their linear combinations could lead to an early diagnostic on biennial bearing (Lauri et al., 2014), no correlations or co-localization could be found in SG family, which was the only one studied for early tree architectural development among the five families considered here. In future work, including the type of bourse shoot within successive floral AS could improve the characterization of genetic variations and their relationships with architectural factors.

### Potential use in breeding of genitors or founders

In a breeding perspective, three descriptors should be combined to ensure regular bearing behavior (i.e., BBI_res_norm_pred, γ^*pred*^ and entropy). Five major QTLs on LG 4, 5, 8, 9, and 10 should be checked and alleles adequately combined in new released materials. Considering the strong evidence of QTLs for these three descriptors, appropriate phenotypes could be targeted with low BBI_res_norm_pred and medium or high γ^*pred*^ at the tree scale with high entropy values avec the axis scale. By contrast, trees with low values of BBI_res_norm_pred, medium or high γ^*pred*^ values and high entropy values at the tree scale could not be observed in the studied populations. As underlined by Samach and Smith (2013), the evolutionary advantage of masting (i.e., synchronicity of flowering at tree and population levels) remains questionable. Our results suggest that flowering synchronicity at the tree level could not be associated with regularity probably because it would lead to over-cropping and major drawbacks in an agronomic context. Knowing if flowering desynchronization has been selected during the apple domestication remains an open question.

This study revealed a complex genetic architecture of flowering habit in apple. The overview of all loci involved in trait variation led us to assess promising individuals and progenitors. X-3259 appeared as the most promising parent whereas “Granny Smith,” which has been phenotypically characterized as a regular phenotype (Lespinasse, 1977), did not cumulate the highest number of favorable alleles. “Coop17” was the most promising founder, estimated to be homozygous for the favorable allele at all QTLs. “Golden Delicious” and “Cox Orange,” widely used as founders in breeding programs (Noiton and Shelbourne, 1992), also carried a relatively high number of favorable alleles. Such reliable overview of the loci involved in bearing habit and estimation of the genotype at major loci is crucial for making relevant choices for breeding. The pedigree-based approach used herein takes the relationships between individuals into account by identity by descent and allows the transmission of alleles to be followed across a pedigree. This approach is particularly relevant for plant species in which varieties are tightly related to each other, which is the case in most crops (Soleimani et al., 2002). In addition, the relative importance of loci and the cumulative effects of small loci should not be overlooked. In this perspective, genomic selection models would be complementary to QTL analyses to evaluate the genetic value of individuals by summing allelic effects at each position of the genome (Kumar et al., 2013; Muranty et al., 2015).

## Author contributions

EC planned and designed the research. AA and BG performed experiments and conducted fieldwork, JBD. performed statistical analyses, AA, Ev, and MB performed QTL detection. JBD, AA, BG, Ev, and EC interpreted the results and wrote the manuscript.

### Conflict of interest statement

Author Marco Bink is currently affiliated with Hendrix Genetics, however this work was completed whilst at a previous affiliation, Biometris, Wageningen University and Research centre, Droevendaalsesteeg. Therefore Marco Bink, and all other authors, have no competing interests to declare. The reviewer PB and handling Editor declared their shared affiliation, and the handling Editor states that the process met the standards of a fair and objective review.
