# Supplementary material for: Predicting Flowering Behavior and Exploring Its Genetic Determinism in an Apple Multi-family Population Based on Statistical Indices and Simplified Phenotyping
Source: Front Plant Sci. 2017 Jun 7;8:858. doi: 10.3389/fpls.2017.00858 (PMC5461300; doi:10.3389/fpls.2017.00858)
Supplement: Supplementary file 1 [file Table1.pdf]

Genetic determinisms of flowering regularity in apple tree: a multi-family QTL detection based on statistical indices extracted from branch sequence analyses

Jean-Baptiste Durand[1,2,†], **Alix Allard**[3,†], **Baptiste Guitton**[3], **Eric van de Weg**[4], **Marco Bink**[5], **Evelyne Costes**[3(*)]

[†] First co-authorship

Corresponding author: Evelyne Costes , costes@supagro.inra.fr

Telephone: +33(0)4 67 61 75 08; fax: +33(0)4 67 61 55 96.

Supplementary Information

Figures

Figure S1. Histograms of the sequence lengths (corresponding to the number of years) for the SG (a), XB (b), HIVW (c), P (d) and N (e) families.

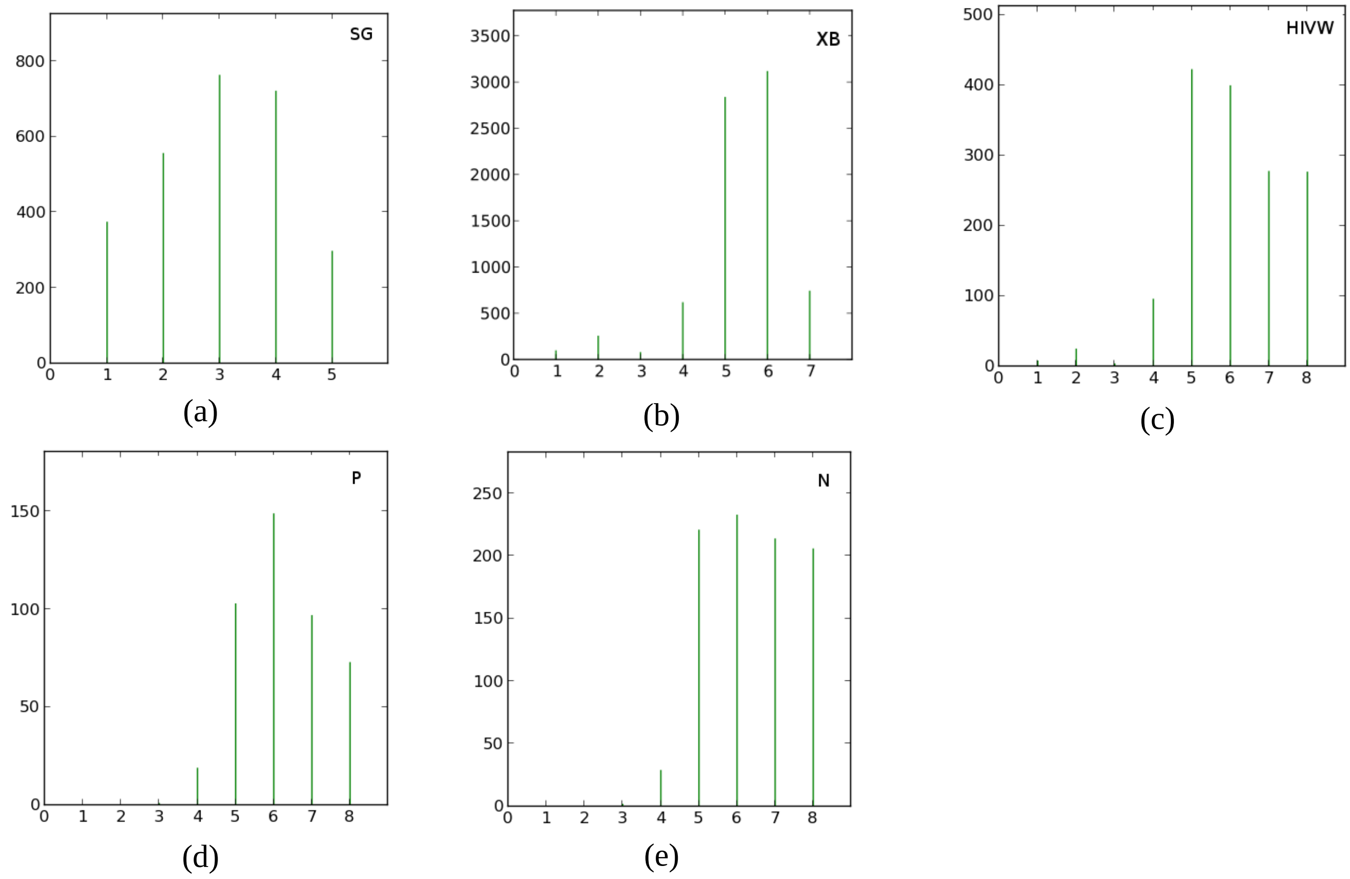

Figure S2. Trace plot of QTL presence along the genome and across iterations for BBI-derived indices, autoregressive coefficients and entropy. The variables displayed are (A) BBI_res_norm_ax, (B) BBI_res_norm_pred, (C) γ^{ax}, (D) γ^{pred}, (E) $\overline{Ent}_g$, (F) $\overline{Ent}_{glmm,g}$. See text for abbreviation meaning. The number of QTL was assigned a Poisson prior with different values (i.e., 5, 10) to assess sensitivity of posterior inference to the prior assumptions. Results for prior mean of 5 are reported only, the other values yielding similar results and inferences.

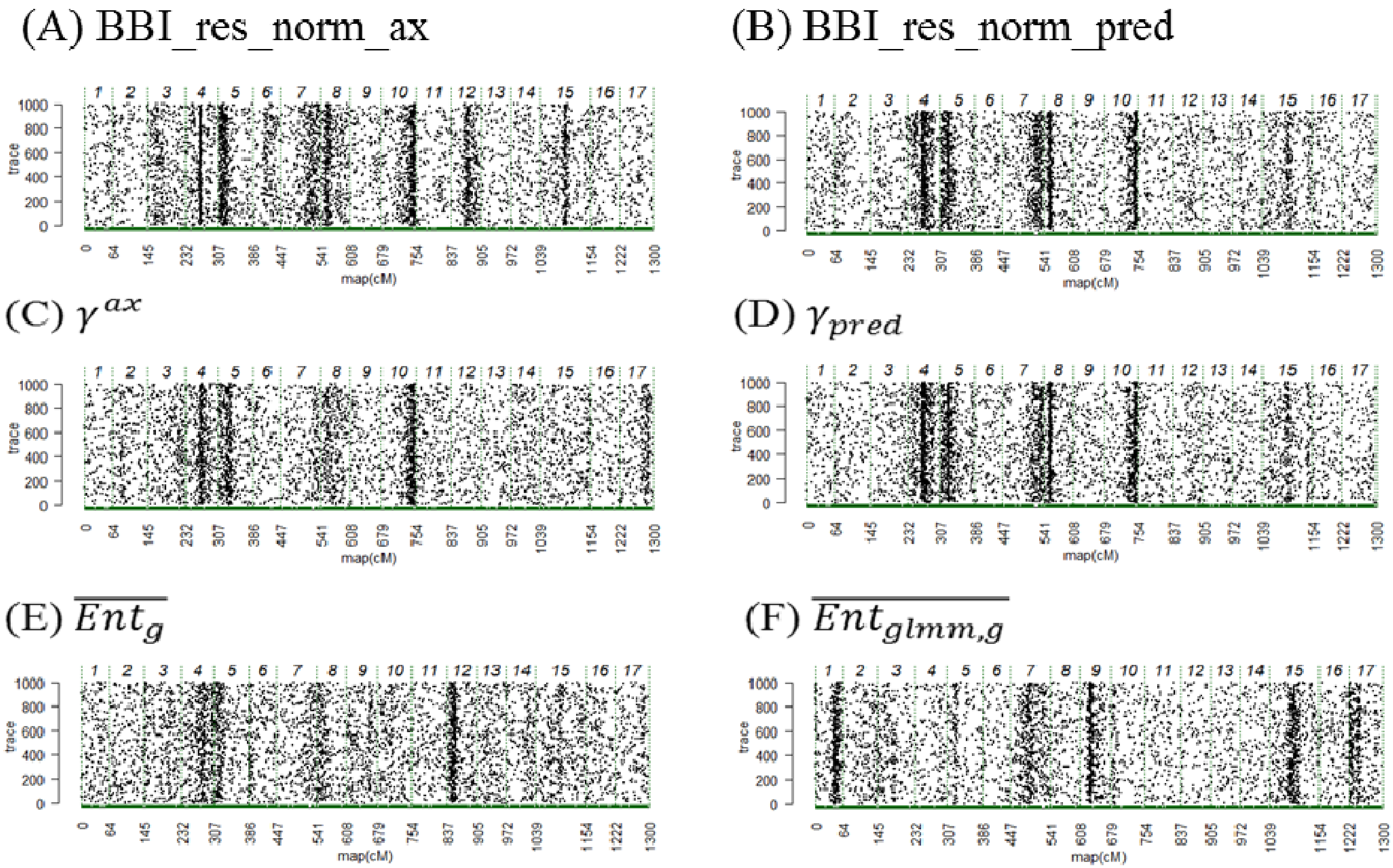

Figure S3. : Posterior probability of QTL position along the genome, the beginning and the end of the chromosomes are represented by vertical dashed lines. The variables displayed are for the genotype x year interactions and the genotype x memory interactions. (A) $\eta_{g,2006}$, (B) $\eta_{g,2008}$, (C) $\theta_{g,00}$, (D) $\theta_{g,01}$, (E) $\theta_{g,10}$, (F) $\theta_{g,11}$,. See text for abbreviation meaning

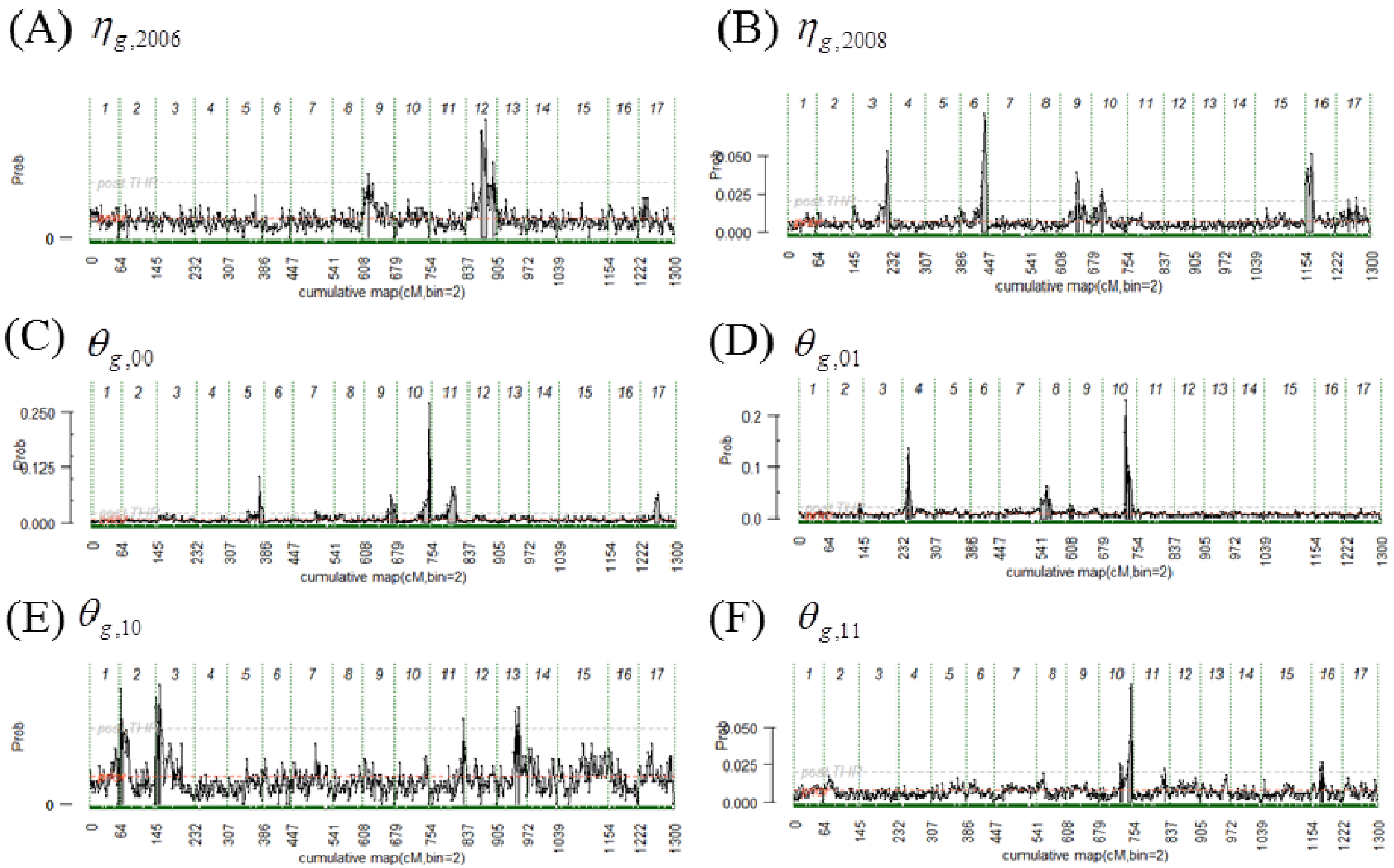

Figure S4. Trace plot of QTL presence along the genome and across iterations for the genotype x year interactions and the genotype x memory interactions. (A) $\eta_{g,2006}$, (B) $\eta_{g,2008}$, (C) $\theta_{g,00}$, (D) $\theta_{g,01}$, (E) $\theta_{g,10}$, (F) $\theta_{g,11}$ .The number of QTL was assigned a Poisson prior with different values (i.e., 5, 10) to assess sensitivity of posterior inference to the prior assumptions. Results for prior mean of 5 are reported only, the other values yielding similar results and inferences.

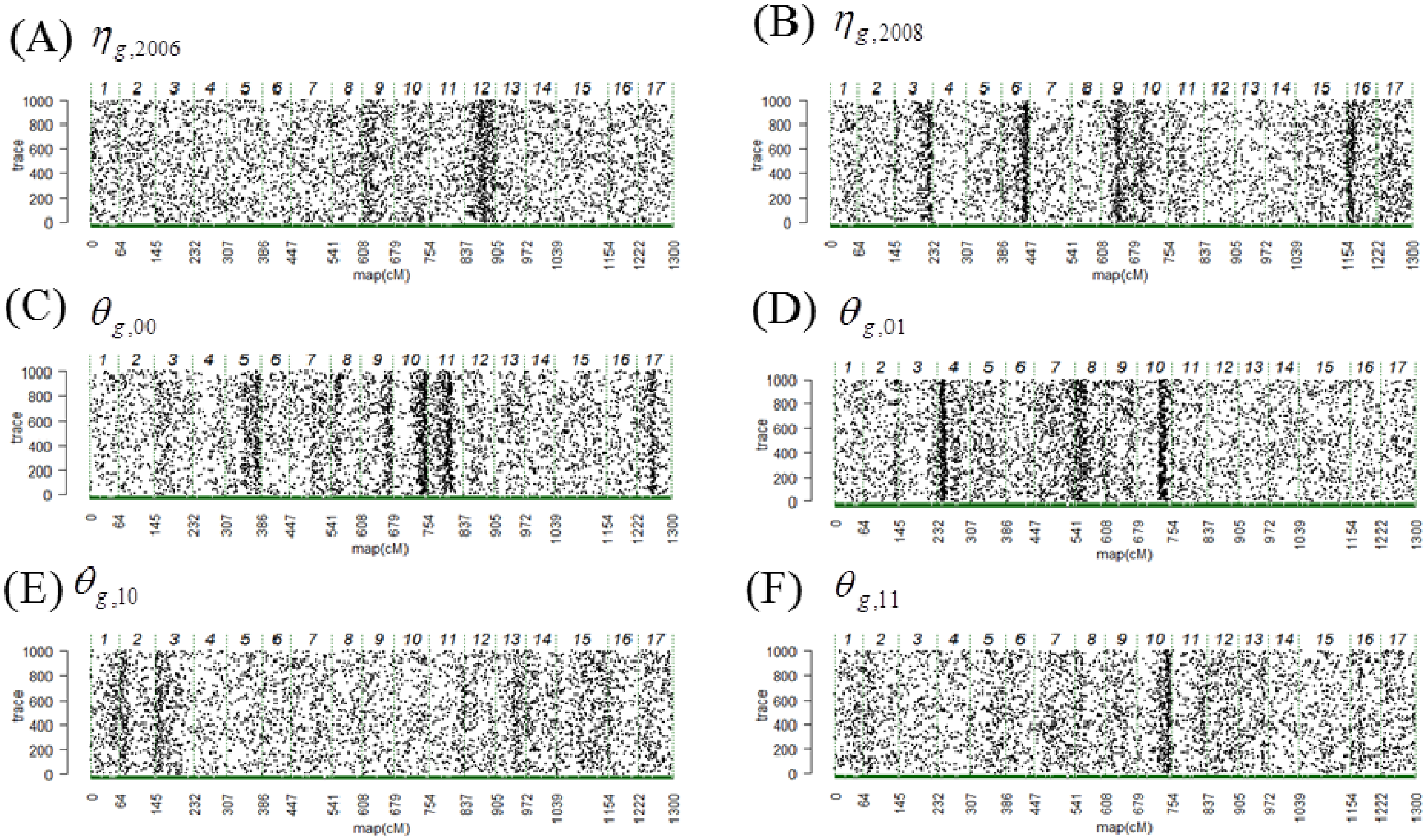

Figure S5. Empirical mean values of BLUPs for genotype and year interactions depending on the site (left) or the family (right).

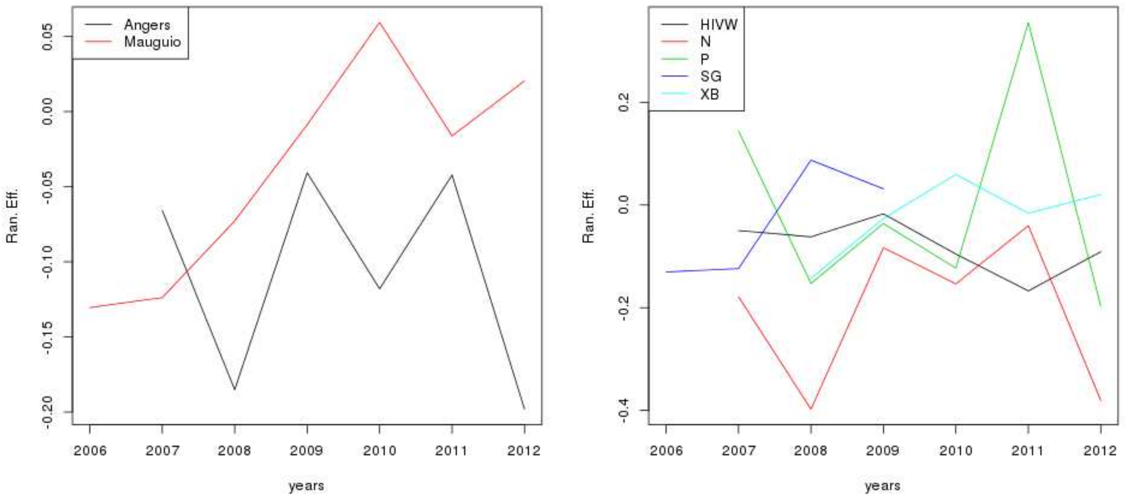

Tables

Table T1. P-values of the ANOVA tests for the effect of the class of bearing behaviours on axis-scale indices in the SG family. B^{ax} is a shorthand for BBI_res_norm_ax. See text for other indices meaning.

Index	$\theta_{g,01}$	$\theta_{g,11}$	$\theta_{g,00}$	$\theta_{g,10}$	$\overline{Ent}_g$	$\overline{Ent}_{glmm,g}$	B^{ax}	γ^{ax}
p-value	7.58e-09	0.127	0.0418	0.895	2.06e-08	0.0143	2e-16	2.75e-09

Table T2. Parameters associated with the QTL detected for the BLUPs of genotype x year interactions and genotype x memories interactions. The first column indicates the variable concerned, the following columns indicate the LG where the QTL is located, 2ln(BF) value at LG scale, 2ln(BF) value at bin scale, the position of the QTL in cM, the position of the QTL peak, its additive effect, the frequency of positive allele and percentage of variance explained, respectively. Only 2lnBF values corresponding to the comparison of a model with 0 QTL to a model with 1 QTL are presented. QTLs that appear in bold are QTL with a strong evidence for presence, i.e. with a 2*lnBF value higher than 5.

	LG	2lnBF_LG	max_2lnBF_bin	pos (cM)	Peak (cM)	add_ef	fq	%var
$\theta_{g,00}$	**10**	**5,4**	**7,7**	**59-76**	**75-76**	**0,22**	**0,66**	**10**
	11	3,2	4,8	36-55	46-47	0,28	0,35	15
$\theta_{g,01}$	4	3,8	6	8-23	16-17	0,16	0,38	5,9
	**10**	**8,1**	**7,3**	**49-68**	**53-54**	**0,24**	**0,21**	**11,8**
$\theta_{g,10}$	no QTL							
$\theta_{g,11}$	10	1,9	4,8	65-76	75-76	0,14	0,43	6,9
$\eta_{g,2006}$	12	2,5	3,6	34-47	44-45	0,58	0,52	14,4
$\eta_{g,2007}$	no QTL							
$\eta_{g,2008}$	6	2,3	4,8	48-61	54-55	0,41	0,6	7,2
$\eta_{g,2009}$	no QTL							
ηg,2010	no QTL							
ηg,2011	no QTL							
ηg,2012	no QTL							

M. Supplementary Description of indices, models and methods

M1/ Reminders: indices for characterization of regular, irregular and alternate bearing genotypes.

It was shown in Durand *et al.* (2013) that genotypes can be categorized in three classes of global bearing habit: regular, irregular and alternate bearing. The genotype clustering relied on two indices computed using the production (number of flowers) $Y_{g,r,\pi,t}$ of tree replication *r* of genotype *g* at place π and year *t*, based on trend model

$$Y_{g,r,\pi,t} = \beta + \beta_\pi + \beta_g + (\alpha + \alpha_\pi + \alpha_g + \xi_{g,r})t + \varepsilon_{g,r,\pi,t} \qquad (A)$$

where β and α are fixed parameters, β_π and α_π are fixed place effect, β_g and α_g are fixed genotype effects and $\xi_{g,r}$ are independent Gaussian random replication effects, with common variance τ_ξ^2.

The BBI_res_norm is dedicated to discrimination between regular and other genotypes. This index is defined as

$$\text{BBI\_norm} = \frac{\sum_r \sum_{t=2}^{T_{g,r}} \left| Y_{g,r,\pi,t} - Y_{g,r,\pi,t-1} \right| / \sum_r (T_{g,r} - 1)}{\sum_r \sum_{t=1}^{T_{g,r}} Y_{g,r,\pi,t} / \sum_r T_{g,r}},$$

$$\text{BBI\_res\_norm} = \frac{\sum_r \sum_{t=2}^{T_{g,r}} \left| \hat{\varepsilon}_{g,r,\pi,t} - \hat{\varepsilon}_{g,r,\pi,t-1} \right| / \sum_r (T_{g,r} - 1)}{\sum_r \sum_{t=1}^{T_{g,r}} Y_{g,r,\pi,t} / \sum_r T_{g,r}}.$$

where $T_{g,r}$ denote the number of measurements for replication *r* of genotype *g*, and $\hat{\varepsilon}_{g,r,\pi,t}$ denote the empirical in model (A). BBI_norm is some variant of the usual BBI with some normalisation of the mean absolute differences in production by the mean production.

From a statistical point of view, alternate bearing – as opposed to regular and irregular bearing – can be characterised through negative correlations between successive values of the detrended series of yields. Such correlations can be assessed by an autoregressive model

$$\varepsilon_{g,r,\pi,t} = (\gamma + \gamma_\pi + \gamma_g)\varepsilon_{g,r,\pi,t-1} + u_{g,r,\pi,t} \qquad (I)$$

where $\varepsilon_{g,r,t}$ is the same residual as in trend model (A), γ is a fixed parameter, γ_π the fixed deviation from γ for place π, γ_g the fixed deviation from γ for genotype *g* and $u_{g,r,t}$ the residual of residual $\varepsilon_{g,r,\pi,t}$ of tree replication *r* of genotype *g* at time *t*. It is assumed variables $u_{g,r,\pi,t}$ are independent and Gaussian with mean 0 and variance ρ^2. The so-called genotype AR coefficient γ_g can be used to discriminate alternate bearing from regular / irregular genotypes. Since BBI_res_norm and γ_g are computed using global flower counts at tree scale $Y_{g,r,\pi,t}$, they are referred to as tree-scale indices.

Additional entropy indices were used to measure synchronicity in flowering. The classical entropy index is based on sequences of AS fates $(F_{g,r,\pi,t,\ell})_{\ell \geq 0}$, $(F_{g,r,\pi,t,\ell} = 0)$ denoting the absence and $(F_{g,r,\pi,t,\ell} = 1)$ the presence of flower for replication *r* of genotype *g* at year *t*, at place π (either “Montpellier SG”, “Montpellier XB” or “Angers”) at location (or AS) ℓ in the tree. It is defined as

$$\overline{Ent}_{g,\pi} = \frac{1}{R_{g,\pi}} \sum_r \left\{ -\frac{1}{\sum_t n_{g,r,\pi,t}} \sum_t n_{g,r,\pi,t} \left(\hat{p}_{g,r,\pi,t,0} \log \hat{p}_{g,r,\pi,t,0} + \hat{p}_{g,r,\pi,t,1} \log \hat{p}_{g,r,\pi,t,1} \right) \right\} \qquad (1)$$

where $R_{g,\pi}$ denotes the number of replications for genotype *g*, $n_{g,r,\pi,t} = n_{g,r,\pi,t,0} + n_{g,r,\pi,t,1}$ the total number of AS for replication *r* of genotype *g* at place π and year *t* and $n_{g,r,\pi,t,i}$ the number of AS with fate *i* (*i*=0, 1). For *i*=0, 1, $\hat{p}_{g,r,\pi,t,i} = n_{g,r,\pi,t,i} / n_{g,r,\pi,t}$ is an estimation of the probability of flowering (*i*=0) v. non-flowering.

Note that the variability in the probability of flowering is partly due to genetic variations, but also to climatic effects that are specific to places π and years *t*. To capture the genetic part of variability only in entropy, we have to eliminate the other effects. Thus, rather than estimating $p_{g,r,\pi,t,i}$ directly using the associated frequency as above, we used some statistical

model for $p_{g,r,\pi,t,i}$. Since $F_{g,r,\pi,t,\ell}$ is a binary variable, approaches based on Generalized Linear Mixed Models (GLMMs) are relevant (Molenberghs and Verbeke, 2006). The following GLMM was considered:

$$\log \frac{p_{g,r,\pi,t,1}}{p_{g,r,\pi,t,0}} = \lambda_\pi + \rho_{\pi,g} + \phi_{\pi,t} + \theta_{\pi,g,t} , \quad (2)$$

where λ_π is the fixed effect of place π (with reference $\lambda_{Montpellier} = 0$), $\rho_{\pi,g}$, $\phi_{\pi,t}$ and $\theta_{\pi,g,t}$ are random effects, assumed to be mutually independent and Gaussian, $\rho_{\pi,g}$ being the effect of genotype *g* at place π , $\phi_{\pi,t}$ the interaction between place π and year *t* treated as a qualitative variable, and $\theta_{\pi,g,t}$ the interaction between genotype *g* at place π and year *t*.

Since $n_{g,r,\pi,t}$ may be very small for some genotypes, maximum likelihood estimation may not converge from a computational point of view. Modelling the log ratio of the probabilities allows the probability of a binary variable (comprised between 0 and 1) to be mapped into ℝ.

The model parameters were estimated by maximum likelihood using the function *glmer* of package *lme4* (Bates *et al.*, 2011). To estimate probabilities $\tilde{p}_{g,r,\pi,t,i}$ corrected from all place-related effets, $\log \frac{\tilde{p}_{g,r,\pi,t,1}}{\tilde{p}_{g,r,\pi,t,0}} = \rho_{\pi,g} + \theta_{\pi,g,t}$ was used in model (2). Then to estimate entropies the $\tilde{p}_{g,r,\pi,t,i}$'s were used in lieu of $p_{g,r,\pi,t,i}$ in equation (1).

M2/ Prediction of fruiting behaviour from axis-scale indices

The above indices were used simultaneously to predict genotype habit from subsamples of AS. In our setting, the habit is summed up by a genotype class among *K*=3 possible classes: {regular, alternate, irregular}. The classes obtained for the SG family in Durand *et al.* (2013) by a clustering procedure, using the total number of flowers $Y_{g,r,t}$ for each replication were

used as reference since they are the only genotypes with known behaviour at tree scale. The issue of class prediction from indices corresponds to the statistical framework of supervised classification. It was addressed using feed-forward neural networks (NNs in short), which essentially are non-linear multivariate regression models (Bishop, 2006, Chapter 5). These models provide probabilities for each genotype to belong to every possible class. The R-based implementation *nnet* was used (Venables & Ripley, 2002). The NN parameters were estimated by maximum likelihood, except a regularisation parameter that must be chosen by a statistical model selection principle. Assessment of the prediction quality obtained by NNs and selection of the regularisation parameter were achieved by out-of-sample validation. The principle was to use a random partition of the genotypes with known habits between learning (50%) and test (50%) genotypes. The learning genotypes were used to estimate the NN parameters, i.e. the mapping between indices and classes. Thus the classes were considered as known for the learning genotypes. The test genotypes were used to predict their classes, as if these were unknown. Since their classes were actually known, an error rate could be computed on the test set (so-called *test error*). This error rate was likely to vary according to the random partition. Thus, 5 partitions were drawn at random and the error rate was averaged over the 5 test sets. We selected the model (i.e. regularisation parameter) minimizing the test error. This method was adapted to perform non-linear regression to simultaneously predict both tree scale indices BBI_res_norm and γ_g from the axis-scale indices. In this case, the parameters were estimated by minimising the mean square error and the regularisation parameter was chosen by maximising the sum (over each index) of square correlations between the true and the predicted indices. The test correlations are referred to as cross-validated correlations.

Literature Cited in Supplementary Information

Bates D, Maechler M, Dai B. 2011. lme4: Linear mixed-effects models using S4 classes. R package version 0.999375-28. [WWW document] URL http://lme4.r-forge.r-project.org [accessed 05 October 2011].

Bishop CM. 2006. Pattern Recognition and Machine Learning. Springer Verlag, 2006.

Molenberghs G., **Verbeke G. 2005**. *Models for discrete longitudinal data*. New York: Springer.

Venables WN, Ripley BD. 2002. *Modern Applied Statistics with S*, 4th edn. New York: Springer.
